# Assessing Microcirculation in Resectable Oesophageal Squamous Cell Carcinoma with Dynamic Contrast-enhanced MRI for Identifying Primary tumour and Lymphatic Metastasis

**DOI:** 10.1038/s41598-018-36929-5

**Published:** 2019-01-15

**Authors:** Yan-li Chen, Yu Jiang, Tian-wu Chen, Rui Li, Xiao-ming Zhang, Fan Chen, Lan Wu, Jing Ou, Jian-qiong Yang

**Affiliations:** 1 Sichuan Key Laboratory of Medical Imaging, and Department of Radiology, Affiliated Hospital of North Sichuan Medical College, 63# Wenhua Road, Nanchong, Sichuan China; 2 Department of Radiology, Southwest Hospital, Army Medical University (Third Military Medical University), Chongqing, China

## Abstract

This study aimed to determine whether dynamic contrast-enhanced MRI (DCE-MRI) derived parameters can identify oesophageal squamous cell carcinoma (SCC) and lymphatic metastasis. Thirty-nine oesophageal SCC patients underwent DCE-MRI. Quantitative parameters including endothelial transfer constant (K^trans^), reflux rate (K_ep_), fractional extravascular extracellular space volume and fractional plasma volume, and semi-quantitative parameters including time to peak (TTP), max concentration, Max Slope and area under concentration-time curve of both oesophageal SCC and normal oesophagus were measured. Mann-Whitney U test revealed that K^trans^ and K_ep_ of oesophageal SCC were higher while TTP was shorter when compared to normal oesophagus (all *P*-values < 0.05); and areas under receiver operating characteristic [ROC] curves displayed that K_ep_ was superior to TTP or K^trans^ for identifying oesophageal SCC (0.903 vs. 0.832 or 0.713). Mann-Whitney U test also demonstrated that K_ep_ was higher and TTP was shorter in patients with lymphatic metastasis when compared to non-metastatic cancer patients (both *P*-values < 0.05), and area under ROC curve also showed that TTP was superior to K_ep_ for predicting lymphatic metastasis (0.696 vs. 0.659). In conclusion, the combination of quantitative and semi-quantitative parameters derived from DCE-MRI can aid in the identification of oesophageal SCC and lymphatic metastasis.

## Introduction

Oesophageal cancer is the eighth most common malignant tumour and is the sixth leading cause of cancer-related death worldwide^1^. Among the various forms, the most prevalent histological type is oesophageal squamous cell carcinoma (SCC)^2^. Early detection of oesophageal SCC and prediction of lymphatic metastasis is essential for timely and effectively treatment, which can potentially be life-saving. Neovascularization provides nourishment for the growth and lymphatic spread of oesophageal SCC. Therefore, a better understanding of the angiogenic behavior of oesophageal SCC may be useful in identifying oesophageal SCC and predicting lymphatic metastasis.

The development of perfusion computed tomography (CT) has made it possible to capture the parameters reflecting the vasculature of oesophageal SCC, facilitating the identification of oesophageal SCC. It has also made lymphatic metastasis more predictable^3,4^. However, perfusion CT is limited in clinical use due to concerns of radiation exposure. Magnetic resonance imaging (MRI) is increasingly used for diagnosing and N staging for oesophageal cancer. This is explained largely by the technical improvements (e.g. breath-hold sequences) and the addition of functional MRI techniques such as diffusion-weighted imaging (DWI) and dynamic contrast-enhanced MRI (DCE-MRI)^5–8^. Due to its noninvasive and non-ionising technique, DCE-MRI is often preferred and is being widely used in studies of malignant tumour including breast cancer, prostate cancer and rectal cancer^9–11^. DCE-MRI not only can visually judge the enhancement of a region of interest and semi-quantitatively characterize tumours by analyzing the signal variation with respect to time, but also can quantitatively evaluate tumours with parameters derived from pharmacokinetic models, which demonstrates the dynamic distribution of gadolinium-related contrast agent in the different compartments of the tumour^12–15^. The two-compartment model of DCE-MRI presumes that gadolinium-related contrast agent exchanges between the extravascular-extracellular space (EES) and the plasma space, and the transfer rate of forward and backward can reflect the permeability of the microvascular^16^. Despite the publication of relevant paper regarding DCE-MRI in oesophageal cancer^7^, the published study aimed to reveal the therapeutic effects with DCE-MRI. To our knowledge, there were no publications regarding the combination of quantitative and semi-quantitative parameters derived from DCE-MRI to identify resectable oesophageal SCC and predicting status of lymphatic metastasis. Therefore, the present study was undertaken to evaluate the feasibility of DCE-MRI for the discrimination of microcirculation differences between oesophageal SCC and the normal oesophagus and between oesophageal SCC with and without lymph node metastasis.

## Materials and Methods

### Patients

The institutional review board of North Sichuan Medical College approved this study, and written informed consent was obtained from each participant before the prospective study. All methods were performed in accordance with the relevant guidelines and regulations.

From February 2016 to October 2017, patients with biopsy-confirmed oesophageal SCC were enrolled into this study according to the following inclusion criteria: (a) the patient did not receive any preoperative tumour-related treatment (e.g. radiotherapy or chemotherapy); (b) the patient had no contraindications for DCE-MRI or surgery; (c) the tumour was considered resectable by endoscopic biopsy and CT^17^; and (d) the quality of the DCE-MRI images was good, suggesting that the motion artifacts resulting from random autonomous movement, breathing, heart pulse and vascular pulse was slight enough for us to perform the data analysis. The exclusion criteria were: (a) the patient had received neoadjuvant radiotherapy and/or chemotherapy before surgery (n = 2); (b) the patient had contraindications for DCE-MRI (e.g. claustrophobia or ferromagnetic metal parts in the patient’s body) or surgery (e.g. medically unable to tolerate general anesthesia and major thoracic surgery) (n = 4); or (c) the quality of the DCE-MRI images was poor (n = 2). The initial population consisted of 47 consecutive patients with biopsy-confirmed oesophageal SCC, 8 of which were excluded. Consequently, this study involved 39 patients (30 men, 9 women; mean age, 64.77 years; age range, 48–76 years).

With regards to the site of the tumours in the enrolled participants, 5.1% (2 of 39) were located at the upper thoracic portion of the oesophagus, 66.7% (26 of 39) were located at the middle thoracic portion, and 28.2% (11 of 39) were located at the lower thoracic portion without oesophagogastric junction invovement. All participants underwent double-contrast barium examinations, endoscopic biopsy, CT and thoracic DCE-MRI examinations before surgery. Subsequently, they were scheduled for radical oesophagectomy with three-field lymphadenectomy. All 39 oesophageal cancers were pathologically diagnosed as SCC, and it was confirmed that the cutting edges of the resected oesophageal segment demonstrated no signs of neoplasia. The time interval between DCE-MRI and surgery was less than 2 weeks (mean, 5.85 days; range, 3–11 days), and none of patients received any preoperative tumour-related treatment. The N stage of tumour was clinically determined according to the postoperative histopathologic examination and American Joint Committee on Cancer criteria^18^. They were categorized as N0, N1, N2 and N3 in 21, 9, 7 and 2 patients, respectively.

### DCE-MRI techniques

DCE-MRI was performed on a 3.0 T superconductive magnet (Discovery MR750; GE Medical Systems, Milwaukee, Wis) for all enrolled patients using 32-channel phased array body coil in the chest region with respiratory and electrocardiogram gating. The patients underwent breath training before the examination and were examined in the supine position. Axial and sagittal T2-weighted sequences with fat saturation were obtained for tumour localization using the following scanning parameters: repetition time (TR)/echo time (TE) of 3000–4000/85–95 ms, field of view (FOV) of 360 mm × 360 mm, matrix of 352 × 352, and slice thickness of 4 mm. Prior to the DCE acquisitions, five consecutive axial three-dimensional spoiled-gradient recalled-echo sequences for liver acquisition with volume acceleration were performed by using TR/TE of 3.3/1.5 ms, FOV of 360 mm × 360 mm, matrix of 256 × 192, and slice thickness of 6 mm with different flip angles of 3°, 6°, 9°, 12°and 15° for determination of pre-contrast T1 values. Subsequently, an axial DCE sequence was performed before and after elbow intravenous injection of 15 ml Gadodiamide (Omniscan; GE Healthcare, Cork, Ireland) of 0.5 mmol/ml; the parameters for this test were arranged as follows: TR/TE of 3.3/1.5 ms, flip angle of 15°, FOV of 360 mm × 360 mm, matrix of 256 × 192, slice thickness of 6 mm, 40 dynamics, temporal resolution of 7 s, and duration of 5 min 4 s. The contrast agent was injected intravenously at the fourth dynamic acquisition using a high pressure injector system (Spectris MR Injector System; Medrad, Pittsburgh, PA, USA), immediately followed by a 20 ml saline flush at a rate of 2.5 ml per second. Based on the published literature^19^, the initial pre-injection dynamic acquisitions of the axial DCE scans would provide the baseline images for generating the time courses when the DCE data analysis was performed.

### Data analysis

**Figure 1:**
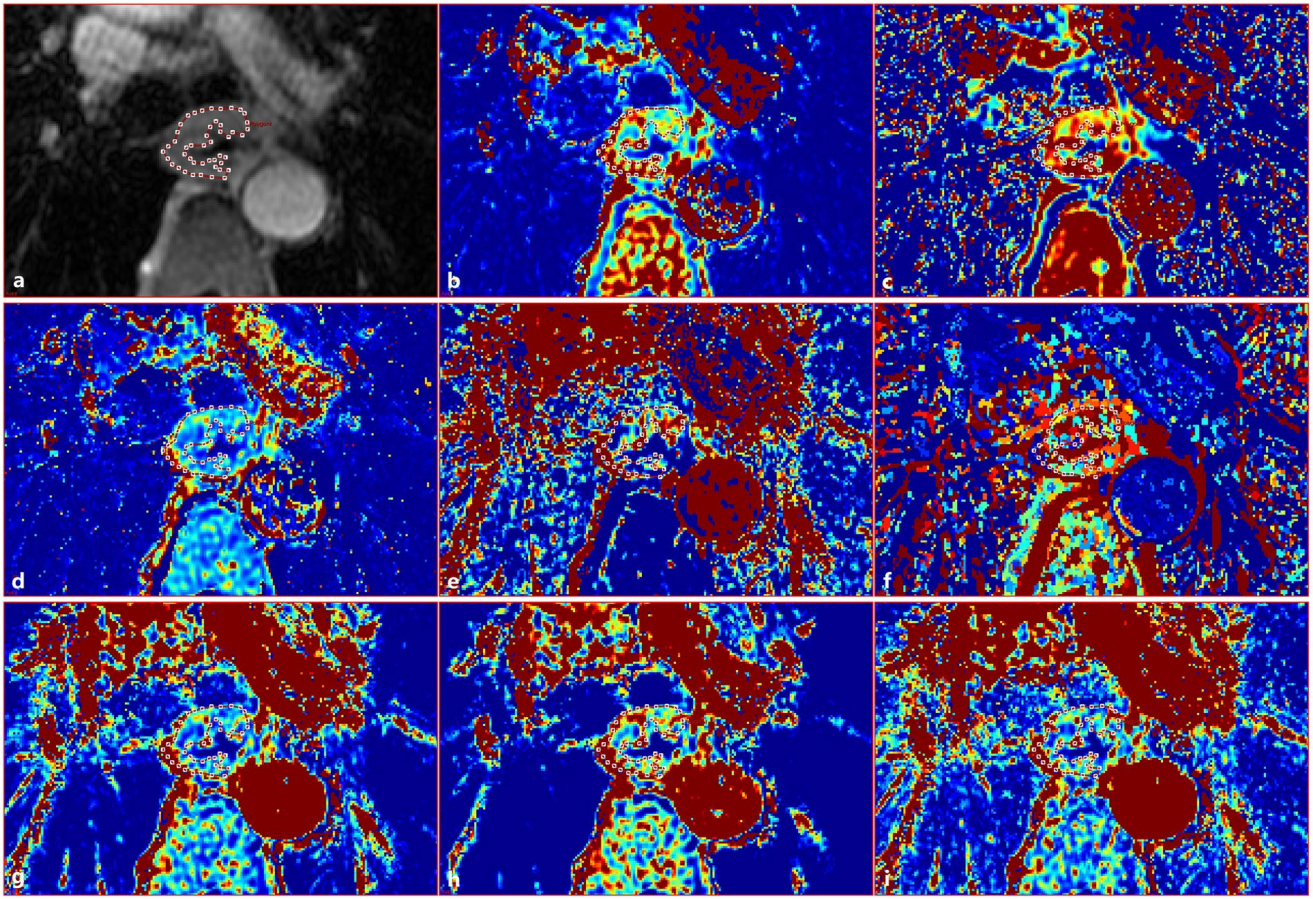
In a 66-year-old man with squamous cell carcinoma in the middle thoracic portion of oesophagus with lymph node metastasis, an irregular region of interest (**a**) for the tumour is drawn within the thickened oesophageal wall to generate the dynamic contrasted-enhanced magnetic resonance imaging derived parameters. Color parametric maps of endothelial transfer constant (**b**), reflux rate (**c**), fractional extravascular extracellular space volume (**d**), fractional plasma volume (**e**), time to peak (**f**), max concentration (**g**), area under the concentration-time curve (**h**), and max slope (**i**) indicate the value of the parameters ranging from high (red) to low (blue). The parameter values correspond to 0.24 ml/min, 0.70 ml/min, 0.36 ml/ml, 0.05 ml/ml, 2.21 min, 1.50 mmol, 4.20 mmol∙min, and 4.31 mmol/min, respectively.

The dynamic data were processed by using the special post-processing software (Omni-Kinetics; GE Healthcare, Bethesda, MD, USA) which provides pharmacokinetic measurement and calculation on a pixel-by-pixel basis. Two radiologists with experience in digestive radiology (Y.L.C. with 4 years of experience, and T.W.C. with 21 years of experience) who were blinded to the pathological results independently performed the data analysis. After the dynamic images were downloaded into this software, motion correction was automatically performed. A T1 mapping was computed from T1-weighted acquisitions with different flip angles (α = 3°, 6°, 9°, 12° and 15°). An arterial input function was extracted by randomly manually drawing a circle region of interest (ROI) with the diameter of 1 cm on the descending aorta. Next, the tumoural ROI was freehand outlined (Fig. 1a) randomly on one maximal section on magnified images, and the area of the ROI was more than 60% of the area of the tumour. On T2WI, the thickened oesophageal wall with a slightly higher signal intensity when compared to normal oesophageal tissue was defined as oesophageal SCC, which could help us in determining the exact boundaries of oesophageal SCC for the drawn of tumoural ROI. The necrotic areas, hemorrhagic areas, intraluminal gas and paraoesophageal fat were excluded based on the conventional magnetic resonance images (axial and sagittal T2-weighted sequences with fat saturation). The derived quantitative parameters including endothelial transfer constant (K^trans^, in ml/min), reflux rate (K_ep_, in ml/min), fractional extravascular extracellular space volume (V_e_, in ml/ml) and fractional plasma volume (V_p_, in ml/ml) were automatically generated on the basis of a two-compartment modified Tofts model^20^. The semi-quantitative parameters including time to peak (TTP, in min), max concentration (MAX Conc, in mmol), Max Slope (in mmol/min) and area under the concentration-time curve (AUC, in mmol·min) were also automatically generated. The previous software automatically derived the parametric maps of K^trans^ (Fig. 1b), K_ep_ (Fig. 1c), V_e_ (Fig. 1d), V_p_ (Fig. 1e), TTP (Fig. 1f), MAX Conc (Fig. 1g), AUC (Fig. 1h) and Max Slope (Fig. 1i) that showed all tissues within the selected processing threshold. The previous process and analyses were repeated for the contiguous another two representative transverse levels. The mean value of each DCE-MRI derived parameter of oesophageal SCC was obtained by averaging the corresponding parameters across the three tumoural ROIs in each patient. To verify the intra-observer reproducibility of the DCE-MRI parameter measurement, measurements of tumoural DCE-MRI derived parameters were repeated by Y.L.C. one month later.

The quantitative and semi-quantitative parameters measurements of DCE-MRI of normal oesophageal wall were performed by using a similar method to the one used for obtaining the measurements of oesophageal SCC except that the ROI of normal oesophageal wall was outlined on magnified images covering more than 50% of the normal oesophageal wall. As reported^21^, a 3 cm proximal and distal margin from the primary tumour was adequate to cover microscopic disease within oesophagus in 94% patients with oesophageal SCC. In this study, we choose the proximal oesophagus above 3 cm of the tumour margin as normal oesophagus except the distal oesophagus above 3 cm of the tumour margin in 2 patients with oesophageal SCC involved the upper thoracic portion of the oesophagus for the previous DCE-MRI derived parameter measurement. In total, 24 cases with sufficient quality of the DCE-MRI images of the margin from the primary tumour were chosen for the measurements of DCE-MRI parameters of normal oesophageal wall in our study.

### Statistical analysis

SPSS statistical package version 13.0 was used for statistical analysis (version 13.0 for Windows, SPSS Inc., Chicago, IL, USA). Data are expressed as mean ± standard deviation.

Inter-observer and intra-observer agreements for the measurements of oesophageal SCC DCE-MRI parameters were evaluated by using the inter-class correlation coefficient (*ICC*). The agreement was defined as excellent (*ICC* > 0.90), good (*ICC* = 0.75–0.90), moderate (*ICC* = 0.5–0.75), or poor (*ICC* < 0.5)^22^. If the inter- and intra-observer agreements of the measurements of oesophageal SCC DCE-MRI parameters were good (*ICC* > 0.75), values of the first measurement by Y.L.C. were regarded as the final pharmacokinetic parameters for the oesophageal SCC. If not, the mean value of the two measurements by Y.L.C. and the measurement by T.W.C. was used as the final values.

We used the Mann-Whitney U test to compare the DCE-MRI derived parameters between oesophageal SCC and normal oesophageal wall or between the statuses of lymphatic metastasis. Statistically significant difference was assigned to less than 0.05. If there were significant differences in the DCE-MRI derived parameters between oesophageal SCC and normal oesophageal wall or between the statuses of lymph node metastasis, then the receiver operating characteristic (ROC) analysis was performed to determine if the cutoff of any DCE-MRI derived parameter could help identify oesophageal SCC and lymph node metastasis.

## Results

### Inter- and intra-observer variability of DCE-derived parameter measurements

**Figure 2:**
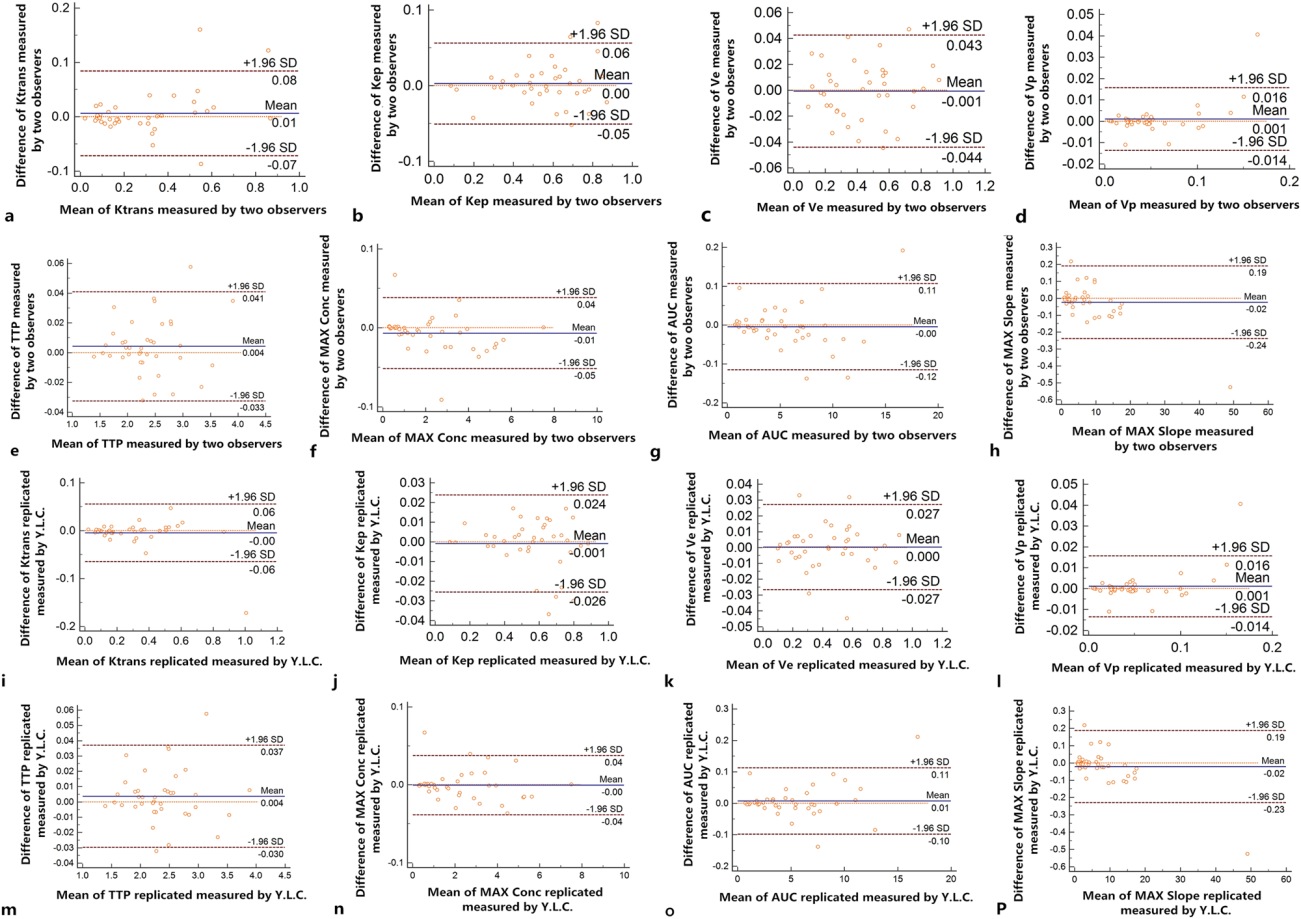
The scatter plots are displaying the inter-observer agreements of K^trans^ (**a**), K_ep_ (**b**), V_e_ (**c**), V_p_ (**d**), TTP (**e**), MAX Conc (**f**), AUC (**g**) and Max Slope (**h**) as well as the intra-observer agreements of K^trans^ (**i**), K_ep_ (**j**), V_e_ (**k**), V_p_ (**l**), TTP (**m**), MAX Conc (**n**), AUC (**o**) and Max Slope (**p**). Notes: K^trans^, endothelial transfer constant; K_ep_, reflux rate; V_e_, fractional extravascular extracellular space volume; V_p_, fractional plasma volume; TTP, time to peak; MAX Conc, max concentration; and AUC, area under the concentration-time curve.

Based on the DCE-MRI derived parameters of oesophageal SCC obtained independently by the two radiologists and repeatedly by Y.L.C., the inter- and intra-observer agreements were both good (*ICC* 95%CI range, both 0.984~0.999; Tables 1 and 2, and Fig. 2). Therefore, the inter- and intra-observer variability was small, and the values of the first measurement by Y.L.C. were used as the final DCE-MRI derived parameters for the subsequent analysis. In addition, the mean diameter of oesophageal SCC on transverse section was 1.42 cm (ranged from 0.63 cm to 1.94 cm).

**Table 1:** Inter-observer variability of dynamic contrast-enhanced magnetic resonance imaging derived parameter measurements.

Dynamic parameter	Difference between two observers	95% Confidence interval	95% Limit of agreement	95% Inter-observer correlation coefficient
Quantitative parameters				
K^trans^	0.006 ± 0.040	−0.072 to 0.084	−0.094 to 0.106	0.9921 (0.9848 to 0.9959)
K_ep_	0.003 ± 0.027	−0.051 to 0.056	−0.066 to 0.071	0.9949 (0.9902 to 0.9973)
V_e_	−0.001 ± 0.022	−0.044 to 0.043	−0.056 to 0.055	0.9924 (0.9854 to 0.9960)
V_p_	0.001 ± 0.008	−0.014 to 0.016	−0.018 to 0.020	0.9918 (0.9844 to 0.9957)
Semiquantitative parameters				
TTP	0.004 ± 0.019	−0.033 to 0.041	−0.043 to 0.051	0.9995 (0.9990 to 0.9997)
MAX Conc	−0.007 ± 0.023	−0.052 to 0.038	−0.065 to 0.051	0.9997 (0.9995 to 0.9999)
AUC	−0.004 ± 0.057	−0.115 to 0.107	−0.147 to 0.138	0.9998 (0.9997 to 0.9999)
MAX Slope	−0.024 ± 0.110	−0.238 to 0.191	−0.299 to 0.252	0.9999 (0.9997 to 0.9999)

Notes: Data are means ± standard deviations. K^trans^, endothelial transfer constant; K_ep_, reflux rate; V_e_, fractional extravascular extracellular space volume; V_p_, fractional plasma volume; TTP, time to peak; MAX Conc, max concentration; and AUC, area under the concentration-time curve.

**Table 2:** Intra-observer variability of dynamic contrast-enhanced magnetic resonance imaging derived parameters measurements.

Dynamic parameters	Differences between two measurements	95% CI	95% Limits of agreement	95% Intra-observer correlation coefficient
Quantitative parameters				
K^trans^	−0.005 ± 0.031	−0.065 to 0.056	−0.082 to 0.073	0.9979 (0.9961 to 0.9989)
K_ep_	−0.001 ± 0.013	−0.026 to 0.024	−0.033 to 0.031	0.9992 (0.9984 to 0.9996)
V_e_	0.001 ± 0.014	−0.027 to 0.027	−0.034 to 0.034	0.9979 (0.9959 to 0.9989)
V_p_	0.001 ± 0.008	−0.014 to 0.016	−0.018 to 0.020	0.9919 (0.9844 to 0.9958)
Semiquantitative parameters				
TTP	0.004 ± 0.017	−0.030 to 0.037	−0.039 to 0.046	0.9995 (0.9991 to 0.9998)
MAX Conc	−0.001 ± 0.019	−0.039 to 0.038	−0.049 to 0.048	0.9998 (0.9995 to 0.9999)
AUC	0.007 ± 0.054	−0.098 to 0.113	−0.128 to 0.142	0.9998 (0.9997 to 0.9999)
MAX Slope	−0.021 ± 0.106	−0.229 to 0.187	−0.288 to 0.246	0.9999 (0.9997 to 0.9999)

Notes: Data are means ± standard deviations. K^trans^, endothelial transfer constant; K_ep_, reflux rate; V_e_, fractional extravascular extracellular space volume; V_p_, fractional plasma volume; TTP, time to peak; MAX Conc, max concentration; AUC, area under the concentration-time curve; and CI, confidence interval.

### DCE-MRI derived parameters for identifying resectable oesophageal SCC

**Figure 3:**
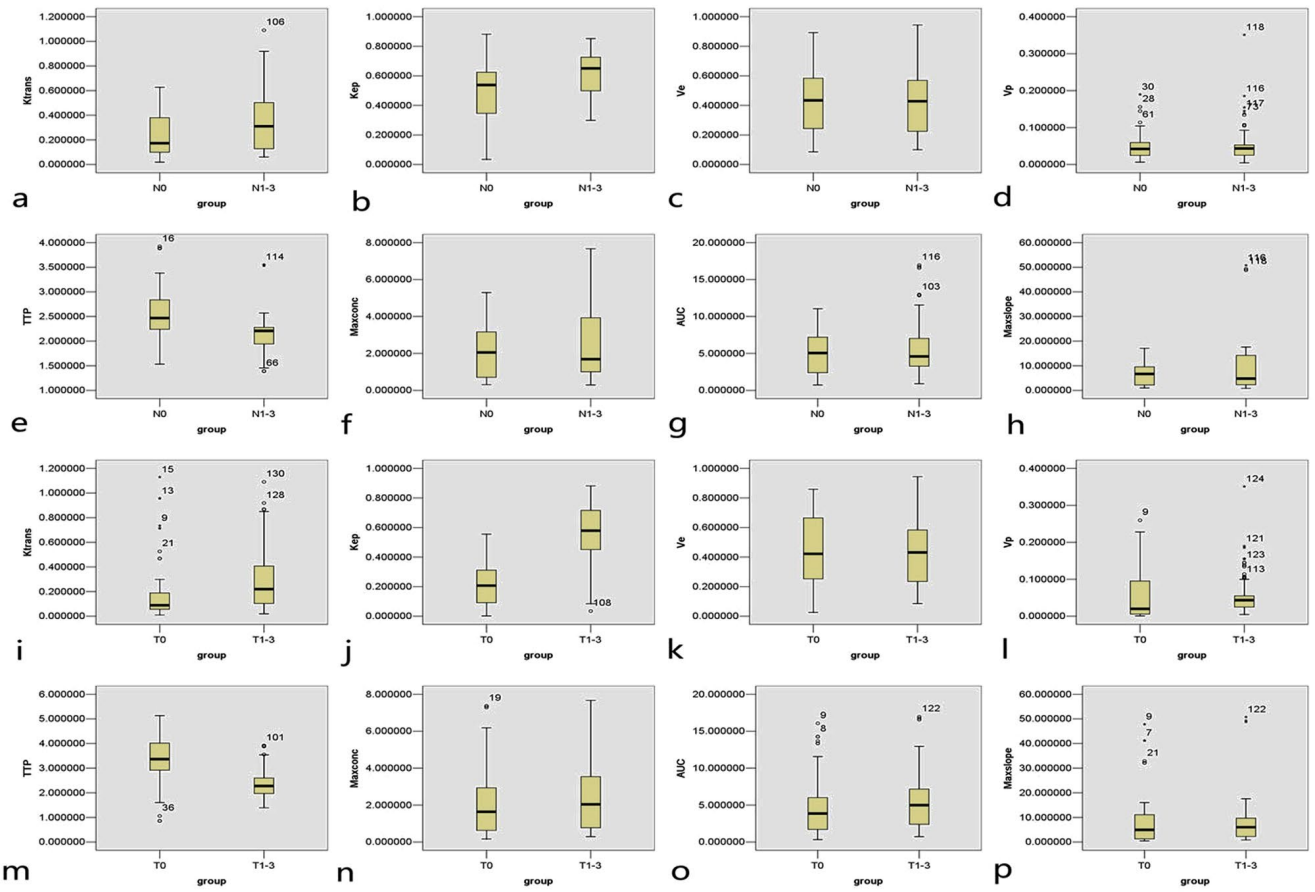
The boxplots are displaying the differences in K^trans^ (**a**), K_ep_ (**b**), V_e_ (**c**), V_p_ (**d**), TTP (**e**), MAX Conc (**f**), AUC (**g**) and Max Slope (**h**) between patients with and without lymph node metastases as well as the differences in K^trans^ (**i**), K_ep_ (**j**), V_e_ (**k**), V_p_ (**l**), TTP (**m**), MAX Conc (**n**), AUC (**o**) and Max Slope (**p**) between oesophageal squamous cell carcinoma (T_1–3_) and normal oesophageal wall (T_0_). Notes: K^trans^, endothelial transfer constant; K_ep_, reflux rate; V_e_, fractional extravascular extracellular space volume; V_p_, fractional plasma volume; TTP, time to peak; MAX Conc, max concentration; and AUC, area under the concentration-time curve.

**Figure 4:**
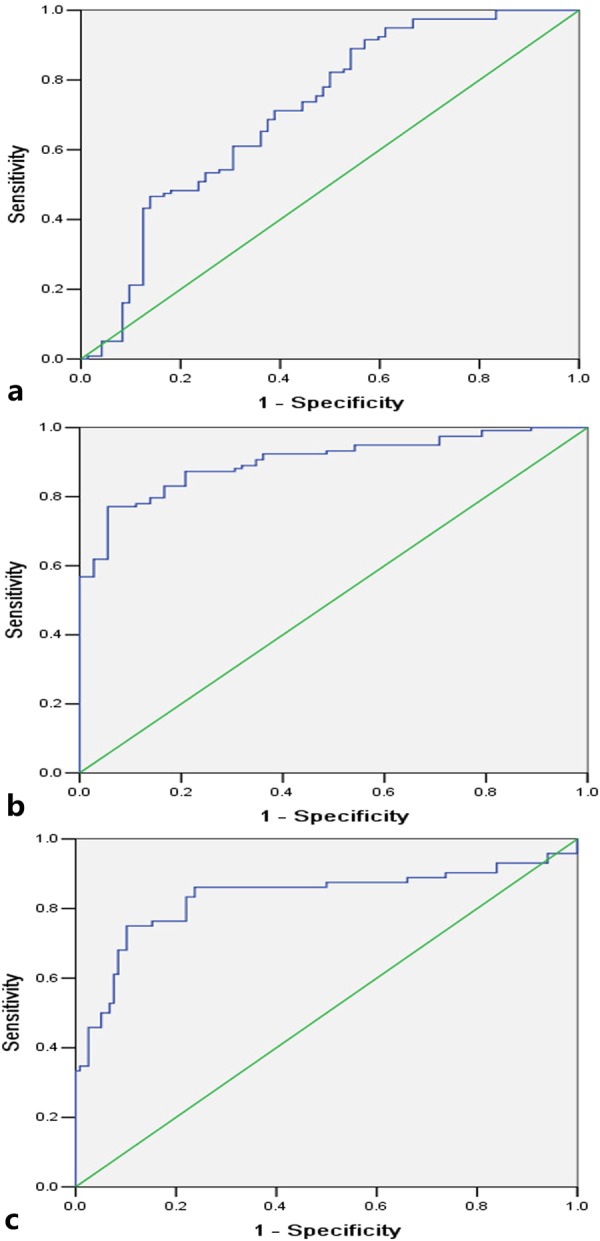
Receiver operating characteristic curves show that endothelial transfer constant cutoff of 0.08 ml/min (**a**), reflux rate cutoff of 0.44 ml/min (**b**), and time to peak cutoff of 2.96 min (**c**) can help differentiate oesophageal squamous cell carcinoma from normal oesophagus.

The Mann-Whitney U tests showed that the quantitative parameters including K^trans^ and K_ep_ of tumour were higher while the semi-quantitative parameter TTP was shorter with significant differences in comparison with normal oesophageal wall (all *P*-values < 0.05) as shown in Table 3 and Fig. 3. The K_ep_ was better than K^trans^ or TTP for oesophageal SCC diagnosis according to the ROC analysis (Table 4; Fig. 4a–c). The Mann-Whitney U tests demonstrated that there were no significant differences in the other quantitative parameters such as V_e_ and V_p_, and semi-quantitative parameters such as MAX Conc, AUC and MAX Slope between the tumour and normal oesophageal wall as shown in Table 3 and Fig. 3 (all *P*-values > 0.05).

**Table 3:** Dynamic contrast-enhanced magnetic resonance imaging derived parameters for identifying resectable oesophageal squamous cell carcinoma.

Dynamic parameters	Carcinoma (n = 39)	Normal oesophagus (n = 24)	*P*-Value
Quantitative parameters			
K^trans^ (ml/min)	0.29 ± 0.23	0.18 ± 0.24	<0.001
K_ep_ (ml/min)	0.56 ± 0.20	0.22 ± 0.15	<0.001
V_e_ (ml/ml)	0.43 ± 0.22	0.43 ± 0.26	0.946
V_p_ (ml/ml)	0.05 ± 0.05	0.05 ± 0.07	0.056
Semiquantitative parameters			
TTP (min)	2.36 ± 0.54	3.42 ± 0.99	<0.001
MAX Conc (mmol)	2.30 ± 1.79	2.21 ± 1.85	0.557
AUC (mmol·min)	5.37 ± 3.65	4.76 ± 3.87	0.141
MAX Slope (mmol/min)	7.65 ± 8.50	8.35 ± 10.18	0.632

Notes: Data are means ± standard deviations. K^trans^, endothelial transfer constant; K_ep_, reflux rate; V_e_, fractional extravascular extracellular space volume; V_p_, fractional plasma volume; TTP, time to peak; MAX Conc, max concentration; and AUC, area under the concentration-time curve.

**Table 4:** Sensitivity, specificity, and the area under receiver operating characteristic (ROC) curve for threshold values to discriminate oesophageal squamous cell carcinoma from normal oesophagus, and tumours with lymph node metastasis from that without nodal disease.

Dynamic parameters	Threshold values	Sensitivity	Specificity	Area under ROC curve
Oesophageal squamous cell carcinoma vs. normal oesophageal wall				
K^trans^	0.08 ml/min	89%	45.8%	0.713
K_ep_	0.44 ml/min	77.1%	94.4%	0.903
TTP	2.96 min	75%	89.8%	0.832
Oesophageal squamous cell carcinoma with vs. without lymph node metastasis				
K_ep_	0.62 ml/min	60%	74.6%	0.659
TTP	2.32 min	71.4%	76.4%	0.696

Notes: K^trans^, endothelial transfer constant; K_ep_, reflux rate; and TTP, time to peak.

### DCE-MRI derived parameters for predicting lymph node metastases

**Figure 5:**
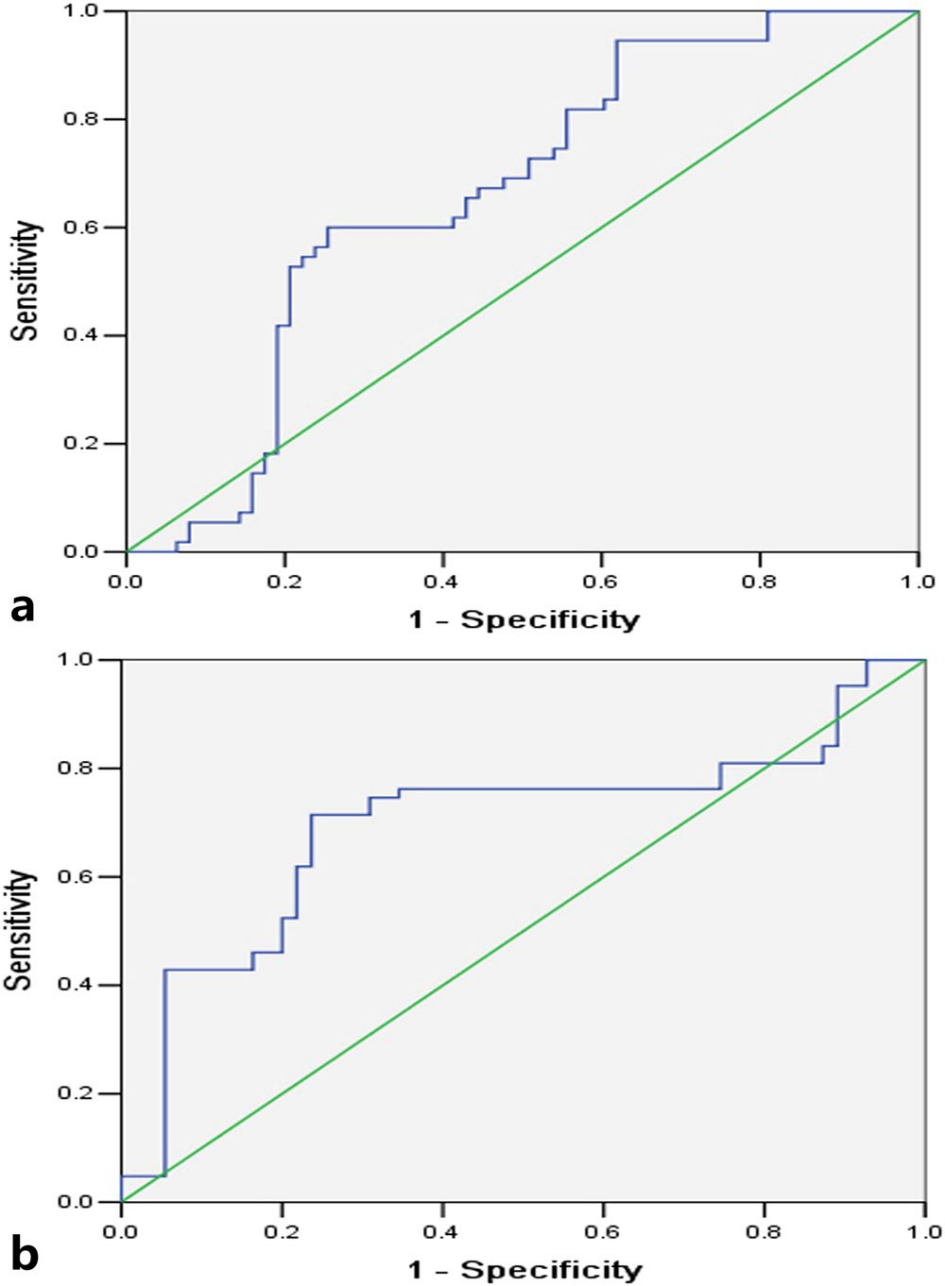
Receiver operating characteristic curves demonstrate that reflux rate cutoff of 0.62 ml/min (**a**), and time to peak cutoff of 2.32 min (**b**) can aid discriminate oesophageal squamous cell carcinoma with and without lymph node metastasis.

According to the Mann-Whitney U tests, there were significant differences in the quantitative parameter K_ep_ and semi-quantitative parameter TTP between tumours with and without lymph node metastases. The K_ep_ was higher whereas the TTP was shorter in tumours of the former as shown in Table 5 and Fig. 3 (both *P*-values < 0.05). In comparison with the K_ep_, the ROC analysis showed that the TTP was better for predicting lymphatic metastasis (Table 4; Fig. 5a,b). The Mann-Whitney U tests demonstrated no significant differences in the other quantitative parameters such as K^trans^, V_e_ and V_p_, and in semi-quantitative parameters such as MAX Conc, AUC and MAX Slope for predicting lymphatic metastasis as shown in Table 5 and Fig. 3 (*P*-values > 0.05).

**Table 5:** Dynamic contrast-enhanced magnetic resonance imaging derived parameters for identifying lymph node metastases (LNM).

Dynamic parameters	With LNM (n = 18)	Without LNM (n = 21)	*P*-Value
Quantitative parameters			
K^trans^ (ml/min)	0.34 ± 0.26	0.25 ± 0.18	0.102
K_ep_ (ml/min)	0.62 ± 0.14	0.50 ± 0.22	0.003
V_e_ (ml/ml)	0.44 ± 0.24	0.43 ± 0.21	0.942
V_p_ (ml/ml)	0.06 ± 0.06	0.05 ± 0.04	0.857
Semiquantitative parameters			
TTP (min)	2.18 ± 0.44	2.51 ± 0.58	<0.001
MAX Conc (mmol)	2.51 ± 2.05	2.12 ± 1.50	0.498
AUC (mmol·min)	5.85 ± 4.26	4.96 ± 2.99	0.566
MAX Slope (mmol/min)	9.00 ± 11.18	6.46 ± 4.93	0.637

Notes: Data are means ± standard deviations. K^trans^, endothelial transfer constant; K_ep_, reflux rate; V_e_, fractional extravascular extracellular space volume; V_p_, fractional plasma volume; TTP, time to peak; MAX Conc, max concentration; and AUC, area under the concentration-time curve.

## Discussion

In our study, we found that several DCE-MRI derived parameters including K^trans^, K_ep_ and TTP displayed significant differences between oesophageal SCC and normal oesophageal wall. In detail, K^trans^ and K_ep_ were higher while TTP was shorter in oesophageal SCC in comparison with normal oesophagus. This finding may be explained by the angiogenesis characteristics of malignancy. Zhao *et al*.^23^ reported that RhoC mRNA expression was mainly located in the cytoplasm of the tumour cells and were higher in oesophageal SCC than in normal oesophagus, and RhoC mRNA expression showed a positive correlation with vascular endothelial growth factor (VEGF) protein levels. Dvorak^24^ demonstrated that VEGF induced endothelial cell division and migration, enhanced microvascular permeability, promoted stromal proteolysis, and reduced endothelial cell apoptosis. Knopp *et al*.^25^ also indicated that the endothelium within tumour microvessels may exhibit increased permeability. These characteristics will allow a more rapid transfer and an accelerated clearance of the contrast agent from the interstitium, ultimately resulting in the higher K^trans^ and K_ep_ and shorter TTP in oesophageal SCC than in normal oesophageal wall.

As shown in our study, the K_ep_ was higher but the TTP was shorter in patients with lymph node metastasis than without that metastasis. Zhao *et al*.^23^ reported that the expression of RhoC mRNA in oesophageal SCC with lymphatic metastasis was significantly higher than without lymph node metastasis, and the expression of VEGF protein in the tumour with lymphatic metastasis was significantly higher than in the tumour without lymphatic metastasis. Zhang *et al*.^26^ also indicated that there was a significant correlation between a high level of VEGF-C expression in oesophageal SCC and lymphatic metastasis. Some published reports indicated that microvessel density of oesophageal SCC with lymph node metastasis was significantly higher than without that metastasis^27–31^. According to the above mentioned reports, the microvascular permeability of increased microvessels was higher in oesophageal SCC with lymph node metastases than without this metastasis, which could lead to the higher K_ep_ and shorter TTP in the cancer with nodal disease.

Because the DCE-MRI derived parameters including K^trans^, K_ep_ and TTP were significantly different between oesophageal SCC and normal oesophageal wall, ROC analysis was performed in this study to determine whether these parameters could be used for differentiating the microcirculation of oesophageal SCC from that of the normal oesophageal wall and applied to the diagnosis of oesophageal cancer. With ROC analysis, the areas under the ROC curves of K^trans^, K_ep_ and TTP were 0.713, 0.903 and 0.832, respectively, which suggested that the K_ep_ was the best parameter for aiding the diagnosis of oesophageal SCC. In addition, the current study showed that the K_ep_ and TTP were significantly different between oesophageal SCC with and without lymph node metastasis, and the areas under the ROC curves of K_ep_ and TTP were 0.659 and 0.696 for determining the microcirculation of oesophageal SCC with lymph node metastasis, respectively. This finding implies that DCE-MRI derived parameters could help identify oesophageal SCC with lymph node metastasis, and TTP could be the better parameter for this purpose.

### Limitations

There were several limitations in our study. Firstly, as squamous cell carcinoma is the most common oesophageal carcinoma worldwide^2^, and we performed this study focusing on assessing the microcirculation of oesophageal SCC rather than that of oesophageal adenocarcinoma with DCE-MRI. We confirmed that microcirculation assessment of oesophageal SCC could help identify this cancer and regional lymphatic metastasis, and we would lead to a new research approach for tumour diagnosis and lymphatic metastasis prediction of oesophageal adenocarcinoma by microcirculation assessment with DCE-MRI. Secondly, the mean values in the ROIs on two-dimensional (2D) image may not reflect spatially rich information within the tumour resulting from the tumour heterogeneity^32^. Thus, prospective studies with heterogeneity analysis on whole-tumour may eliminate the confounding effect in ROI average studies in the future. Thirdly, one of the criteria for the study was that the quality of the DCE-MRI images was good, however this criteria is a little vague. Fourthly, we did not explore the effects of the location of the tumours, and the gender and age of the samples on the accuracy of the findings in this study. We will perform the relevant study in the future.

## Conclusion

The quantitative and semi-quantitative parameters derived from DCE-MRI may potentially be helpful for determining the microcirculation within oesophageal SCC and predicting the status of lymphatic metastasis. The quantitative parameter K_ep_ could be the optimal parameter for identifying oesophageal SCC. The semi-quantitative parameter TTP could be more suitable for predicting lymph node metastasis. We hope that the findings in our study will be helpful for the identification and predication of lymphatic metastasis for treatment decision making.

## References

[1] Ferlay J (2015). Cancer incidence and mortality worldwide: sources, methods and major patterns in GLOBOCAN 2012. Int. J. Cancer..

[2] Torre, L. A. *et al*. Global cancer statistics, 2012. CA Cancer *J. Clin*. **65**, 87–108 (2015). 10.3322/caac.21262 25651787

[3] Chen TW (2009). Quantitative assessment of first-pass perfusion of oesophageal squamous cell carcinoma using 64-section MDCT: initial observation. Clin. Radiol..

[4] Song T (2012). Esophageal squamous cell carcinoma: assessing tumor angiogenesis using multi-slice CT perfusion imaging. Dig. Dis. Sci..

[5] van Rossum PS (2013). Imaging strategies in the management of oesophageal cancer: what’s the role of MRI?. Eur. Radiol..

[6] Giganti F (2016). Prospective comparison of MR with diffusion-weighted imaging, endoscopic ultrasound, MDCT and positron emission tomography-CT in the pre-operative staging of oesophageal cancer: results from a pilot study. Br. J. Radiol..

[7] Oberholzer K (2008). Assessment of tumor microcirculation with dynamic contrast-enhanced MRI in patients with esophageal cancer: initial experience. J. Magn. Reson. Imaging.

[8] Lei J (2015). Preliminary study of IVIM-DWI and DCE-MRI in early diagnosis of esophageal cancer. Eur. Rev. Med. Pharmacol. Sci..

[9] Ryu JK, Rhee SJ, Song JY, Cho SH, Jahng GH (2016). Characteristics of quantitative perfusion parameters on dynamic contrast-enhanced MRI in mammographically occult breast cancer. J. Appl. Clin. Med. Phys..

[10] Gao P, Shi C, Zhao L, Zhou Q, Luo L (2016). Differential diagnosis of prostate cancer and noncancerous tissue in the peripheral zone and central gland using the quantitative parameters of DCE-MRI: A meta-analysis. Medicine (Baltimore).

[11] Shen FU, Lu J, Chen L, Wang Z, Chen Y (2016). Diagnostic value of dynamic contrast-enhanced magnetic resonance imaging in rectal cancer and its correlation with tumor differentiation. Mol. Clin. Oncol..

[12] El Khouli RH (2009). Dynamic contrast-enhanced MRI of the breast: quantitative method for kinetic curve type assessment. AJR Am. J. Roentgenol..

[13] Engelbrecht MR (2003). Discrimination of prostate cancer from normal peripheral zone and central gland tissue by using dynamic contrast-enhanced MR imaging. Radiology.

[14] Jackson A, O’Connor JP, Parker GJ, Jayson GC (2007). Imaging tumor vascular heterogeneity and angiogenesis using dynamic contrast-enhanced magnetic resonance imaging. Clin. Cancer Res..

[15] Oostendorp M, Post MJ, Backes WH (2009). Vessel growth and function: depiction with contrast-enhanced MR imaging. Radiology.

[16] Yankeelov TE, Gore JC (2009). Dynamic contrast enhanced magnetic resonance imaging in oncology: Theory, data acquisition, analysis, and examples. Curr. Med. Imaging Rev..

[17] Ajani JA (2015). Esophageal and esophagogastric junction cancers, version 1.2015. J. Natl. Compr. Canc. Netw..

[18] Edge, S. B. *et al*. AJCC Cancer Staging Manual (7th ed) 103–115 (Springer, 2010).

[19] Chang EY (2008). The evaluation of esophageal adenocarcinoma using dynamic contrast-enhanced magnetic resonance imaging. J. Gastrointest. Surg..

[20] Tofts PS (1999). Estimating kinetic parameters from dynamic contrast-enhanced T(1)-weighted MRI of a diffusable tracer: standardized quantities and symbols. J. Magn. Reson. Imaging.

[21] Gao XS (2007). Pathological analysis of clinical target volume margin for radiotherapy in patients with esophageal and gastroesophageal junction carcinoma. Int. J. Radiat. Oncol. Biol. Phys..

[22] Koo TK, Li MY (2016). A guideline of selecting and reporting intraclass correlation coefficients for reliability research. J. Chiropr. Med..

[23] Zhao ZH, Tian Y, Yang JP, Zhou J, Chen KS (2015). RhoC, vascular endothelial growth factor and microvascular density in esophageal squamous cell carcinoma. World. J. Gastroenterol..

[24] Dvorak HF (2002). Vascular permeability factor/vascular endothelial growth factor: a critical cytokine in tumor angiogenesis and a potential target for diagnosis and therapy. J. Clin. Oncol..

[25] Knopp MV, von Tengg-Kobligk H, Choyke PL (2003). Functional magnetic resonance imaging in oncology for diagnosis and therapy monitoring. Mol. Cancer Ther..

[26] Zhang W (2012). Expression and significance of vascular endothelial growth factor C from multiple specimen sources in esophageal squamous cell carcinoma. Int. J. Biol. Markers.

[27] Igarashi M (1998). The prognostic significance of microvessel density and thymidine phosphorylase expression in squamous cell carcinoma of the esophagus. Cancer.

[28] Ding MX, Lin XQ, Fu XY, Zhang N, Li JC (2006). Expression of vascular endothelial growth factor-C and angiogenesis in esophageal squamous cell carcinoma. World. J. Gastroenterol..

[29] Saad RS, Lindner JL, Liu Y, Silverman JF (2009). Lymphatic vessel density as prognostic marker in esophageal adenocarcinoma. Am. J. Clin. Pathol..

[30] Sakurai T (2014). Endoglin (CD105) is a useful marker for evaluating microvessel density and predicting prognosis in esophageal squamous cell carcinoma. Anticancer Res..

[31] Chen B (2014). The prognostic implications of microvascular density and lymphatic vessel density in esophageal squamous cell carcinoma: Comparative analysis between the traditional whole sections and the tissue microarray. Acta. Histochem..

[32] Huang B, Chan T, Kwong DL, Chan WK, Khong PL (2012). Nasopharyngeal carcinoma: investigation of intratumoural heterogeneity with FDG PET/CT. AJR Am. J. Roentgenol..

